# Exploring exercise-driven inhibition of pyroptosis: novel insights into treating diabetes mellitus and its complications

**DOI:** 10.3389/fendo.2023.1230646

**Published:** 2023-10-04

**Authors:** Nan Li, Liang Zhang, Xintang Wang, Yue Zhou, Lijing Gong

**Affiliations:** ^1^ Department of Exercise Physiology, Beijing Sport University, Beijing, China; ^2^ School of Strength and Conditioning Training, Beijing Sport University, Beijing, China; ^3^ China Institute of Sport and Health Science, Beijing Sport University, Beijing, China; ^4^ Key Laboratory of Physical Fitness and Exercise, Ministry of Education, Beijing Sport University, Beijing, China

**Keywords:** exercise, diabetes mellitus, complications, pyroptosis, therapy

## Abstract

Diabetes mellitus (DM) and its complications are important, worldwide public health issues, exerting detrimental effects on human health and diminishing both quality of life and lifespan. Pyroptosis, as a new form of programmed cell death, plays a critical role in DM and its complications. Exercise has been shown to be an effective treatment for improving insulin sensitivity or preventing DM. However, the molecular mechanisms underlying the effects of exercise on pyroptosis-related diseases remain elusive. In this review, we provided a comprehensive elucidation of the molecular mechanisms underlying pyroptosis and the potential mechanism of exercise in the treatment of DM and its complications through the modulation of anti-pyroptosis-associated inflammasome pathways. Based on the existing evidence, further investigation into the mechanisms by which exercise inhibits pyroptosis through the regulation of inflammasome pathways holds promising potential for expanding preventive and therapeutic strategies for DM and facilitating the development of novel therapeutic interventions.

## 1 Introduction

Diabetes mellitus (DM) is a chronic and progressive disease that is increasing in frequency at an unprecedented rate. Approximately 537 million individuals worldwide are afflicted by diabetes, with a projected prevalence of over 625 million by 2045 (1). Currently, alterations in environment and lifestyle factors such as diet, being overweight and physical inactivity contributed to the increasing number of the eventuality of DM and its associated complications (2). DM and its complications, comprising cardiovascular disease, neuropathy, nephropathy, and retinopathy, are widely recognized for their connection to low-grade chronic inflammation (3–7). Thus, it is essential to understand the regulatory mechanisms of DM-induced organ damage, thereby alleviating the considerable health and economic burden imposed by DM.

Pyroptosis is a form of programmed necrotic cell death, which induces cell swelling and membrane rupture, releases cytosolic contents, and provokes inflammatory reactions (8). An increasing number of studies have confirmed that inflammasomes activate inflammatory caspases, which promote the maturation of proinflammatory molecules, notably IL-1β and IL-18, thereby eliciting immune responses and instigating pyroptosis (9). In recent years, there has been mounting evidence indicating the substantial role of pyroptosis in the progression of diverse ailments, including infectious diseases, nervous system-related disorders, atherosclerosis, tumors, and several other diseases (10–13). DM and its complications are also associated with pyroptosis, and inhibition of that has been shown to be an attractive strategy for delaying disease development (14–16).

Among the factors influencing DM, lifestyle exerts the most pronounced impact on disease progression. Exercise as a valid strategy in the non-pharmacological intervention of lifestyle, holds the potential to alleviate the therapeutic burdens associated with DM and its complications. Targeted anti-inflammatory therapy has been proven for preventing and treating diabetes. Recent studies have demonstrated that exercise could inhibit pyroptosis and proinflammatory cytokines release (17, 18). Moreover, aerobic exercise could ameliorate obesity-induced inflammation and vascular dysfunction by suppressing NLR family pyrin domain containing 3 (NLRP3) inflammasome, concomitantly reducing the levels of caspase-1 and IL-1β (19, 20).

This review outlines the latest progress in the mechanisms underlying pyroptosis, its pivotal role in DM, and related metabolic diseases. We further discuss the potential impact of exercise on regulating pyroptosis, aiming to novel therapeutic strategies targeting the inflammasome for more effective treatment of diabetes and its complications.

## 2 Pyroptosis and its mechanisms

### 2.1 The history of pyroptosis

For decades, apoptosis has been regarded as the predominant, programmed pathway of cell death. Apoptosis is a death triggered by pathological stimuli, which is typically the immunologically silent form of cell death. It is characterized by cell shrinking, chromatin condensation, nuclear fragmentation, and plasma membrane blebbing (21). In 2000, Brennan and Cookson made a noteworthy discovery that cell death occurs in macrophages after infection with Salmonella differs noticeably from conventional apoptosis (22). Although both apoptosis and pyroptosis are forms of cell death, pyroptosis relies on caspase-1 activity. Plasma membrane rupture, the release of cytosolic materials, and DNA fragmentation are its defining features (23). Most importantly, pro-inflammatory cytokines like interleukin‐1β (IL-1β), and IL-18 are rapidly released to the extracellular space and induce inflammation (9).

### 2.2 Mechanism of pyroptosis

**Figure 1:**
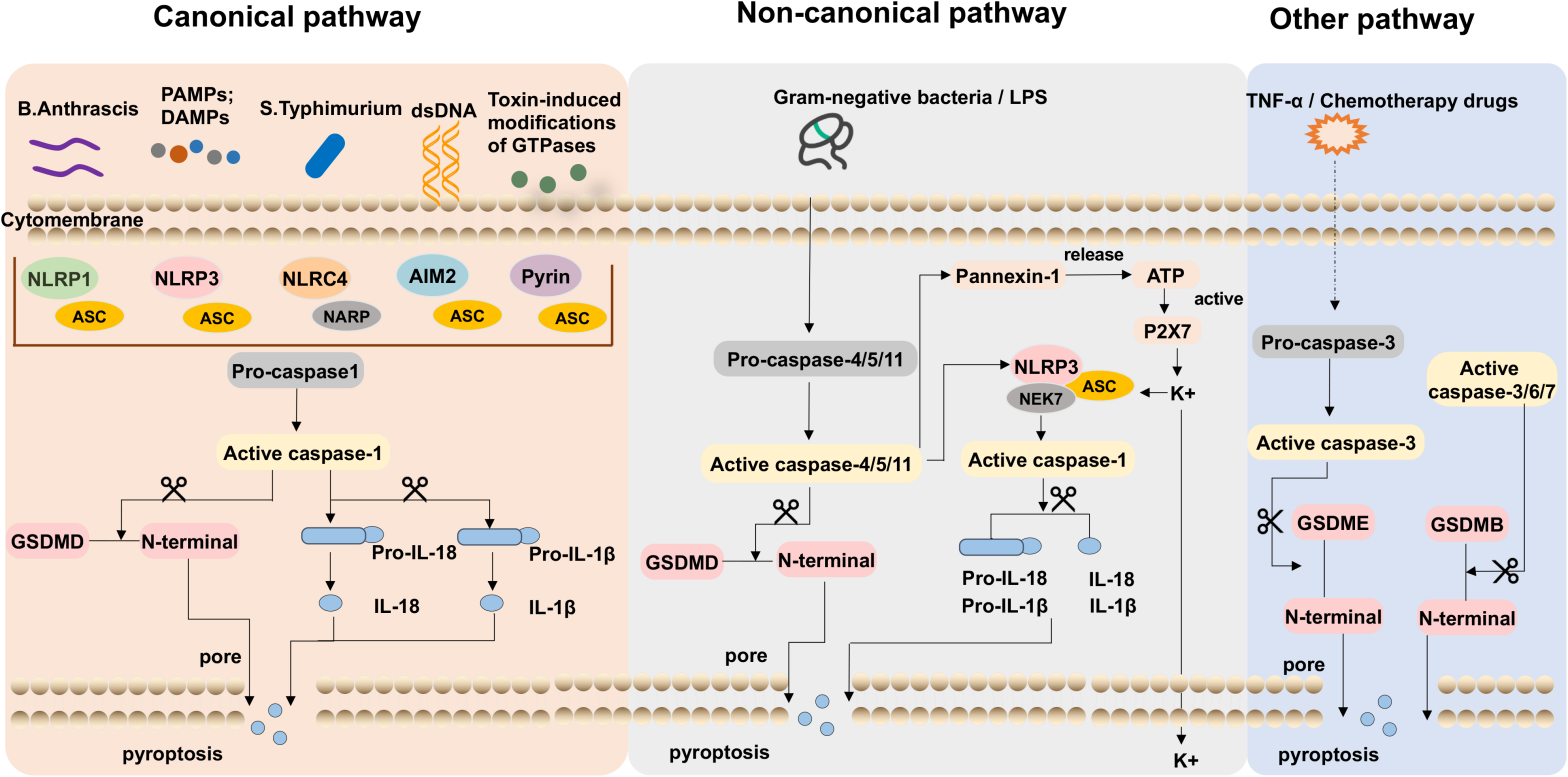
The typical pathways of pyroptosis. In the canonical pathway, including danger-associated molecular patterns (DAMPs) or pathogen-associated molecular patterns (PAMPs) activated, inflammasomes were identified and activated caspase-1, which cleaves gasdermin D (GSDMD) and activates pro-IL-1β and pro-IL-18. The active IL-1β/IL‐18 is released to the oligomeric pores and the N-terminal fragment (GSDMD-NT) inserts into the cell membrane amplifying the inflammatory response. LPS of gram-negative bacteria induces caspase-4/5/11 activation was the non-canonical pathway, it leads to the cleavage of GSDMD and Pannexin-1. GSDMD-NT leads to pore formation on the cell membrane and the release of inflammatory mediators and pyroptosis. The channels Pannexin-1 release cellular ATP and open channel P2X7 resulting in potassium ion efflux. In the other inflammasome pathway, GSDME can be cleaved by caspase-3 and promote cell death.

Pyroptosis is a programmed cell death mechanism that is important for the body’s innate immune defense against infection and inflammation-induced tissue damage, which is mainly executed by gasdermin D (GSDMD). Activated inflammatory caspases cleave GSDMD to release its active fragment, the N-terminal domain of GSDMD (GSDMD-NT), which aggregates on the cell membrane and forms visible pores under electron microscopy, resulting in rapid release of inflammatory cytokines such as IL-1β and IL-18, and causes immune and inflammatory responses (24). Consequently, the GSDMD-NT possesses the capability to induce pyroptosis (9, 25). The body’s innate immune defense against infection and tissue damage is largely dependent on the pyroptosis process, and it has been extensively linked to various inflammatory diseases when dysregulated. Hence, maintaining a delicate balance between inflammatory injury and the healthy immune response to pyroptosis becomes crucial. Several signaling pathways have been described to participate in pyroptosis for now: the caspase-1-dependent canonical pathway, the noncanonical pathway involving caspase-4/5/11, and the newly discovered caspase-3-dependent pathways (**Figure 1**).

#### 2.2.1 The canonical pathway

The classical pathway of inflammasome activation involves the recognition of danger-associated molecular patterns (DAMPs) or pathogen-associated molecular patterns (PAMPs) by pattern recognition receptors (PRRs), which active the inflammasome and the induction of pyroptosis (26, 27). In this pathway, PRRs, including toll-like receptors (TLRs) and NOD-like receptors (NLRs), detect the presence of DAMPs or PAMPs. These molecular patterns can arise from various sources, including cellular damage or invading microorganisms. Upon recognition, PRRs initiate intracellular signaling cascades, leading to the formation of multimolecular complexes (28–31). These multimolecular complexes serve as a platform for the assembly of the inflammasome, which is composed of multiple proteins, including TLRs, NLRs, and AIMs-like receptors (32–34).

A series of inflammasomes, including NLRP1, NLRP3, NLRC4, NLRP6, and AIM2, have been identified later by researchers (35–37). Once the inflammasome is assembled, procaspase-1 undergoes autocleavage, resulting in the generation of active caspase-1 (38). Caspase-1 is a key execution role in pyroptosis. On the one hand, it cleaves GSDMD, leading to the release of GSDMD-NT, which inserts into the cell membrane, forming nonselective pores with inner diameters of 10-14 nm, leading to osmotic imbalances, cell swelling, and pyroptosis. On the other hand, caspase-1 converts IL-1β/IL-18 precursors into mature forms. These active IL-1β/IL‐18 molecules are subsequently released into the pyroptotic cell’s membrane-bound oligomeric pores, amplifying the inflammatory response (24, 39, 40).

#### 2.2.2 The non-canonical pathway

In non-classical inflammasome signaling pathways, the activation of procaspase-4/5/11 by lipopolysaccharide (LPS) plays a central role. Upon activation, procaspase-4/5/11 triggers pyroptosis-mediated cell death (41). LPS can directly activate procaspase-4/5/11, which occurs independently of the inflammasome complex. Once procaspase-4/5/11 binds to LPS, it cleaves GSDMD into GSDMD-NT and leads to pore formation on the cell membrane (42, 43). Moreover, it causes K^+^ to be effluxed, leading to NLRP3 inflammasome to assemble, and eventually resulting in pyroptosis (25). Nevertheless, caspase-4/5/11 could not directly cleave pro-IL-1β/IL-18. In certain cells, it plays an indirect role in mediating the maturation and secretion of IL-1β/IL-18 by activating the NLRP3 inflammasome and subsequent caspase-1 activation. Notably, the activation of caspase-4/5/11 could activate the channel Pannexin-1, which releases cellular ATP. Subsequently, this events prompts the opening of the cytosolic channel P2X7, culminating in potassium ion efflux, thereby triggering NLRP3-mediated pyroptotic cell death (44, 45). The non-classical inflammasome pathway involving procaspase-4/5/11 provides an alternative mechanism for inducing pyroptosis and initiating inflammatory responses. It highlights the diverse pathways and players involved in regulating immune responses and maintaining tissue homeostasis.

#### 2.2.3 The additional caspase pathways

Recent research has demonstrated that caspase-3 is essential for the cleavage of activated gasdermin E (GSDME). Activation of caspase-3 results in its targeting and cleavage of GSDME, leading to the release of N-terminal and C-terminal fragments. GSDME-NT has the ability to bind to the cell membrane and assemble into oligomeric structures. Furthermore, it engenders the formation of pores within the cell swelling, the efflux of cellular contents, and inducing pyroptosis (46, 47). In a recent study, it was found that gasdermin B (GSDMB), which is required for the cleavage of GSDMD in non-canonical pyroptosis, directly interacts with the CARD domain of caspase-4 to increase caspase-4 activity (48). In addition, it has been found that the capacity of GSDMB to create membrane pores may be compromised when its N-terminal domain is cleaved by caspase-3/6/7 (49).

## 3 Mechanisms involved in pyroptosis and its impact on diabetic complications

Pyroptosis plays a crucial role in innate immune defense against microbial infection and tissue damage from excessive inflammation. Substantial evidence supports the development and progression of diabetes and its complications are strongly associated with inflammasomes and activation of various pro-inflammatory. Recent research has provided compelling evidence establishing a link between pyroptosis in β-cells and the pathogenesis of DM (50). Notably, the NLRP3 inflammasome, in particular, has been implicated in β-cell pyroptosis and insulin resistance (51). Carlos D et al. found that NLRP3 activation by mitochondrial DNA (mDNA) results in triggering caspase-1-dependent IL-1β production, thereby contributing to the development of type 1 diabetes mellitus (T1DM) (52). Moreover, thioredoxin-interacting protein (TXNIP), a protein connected to insulin resistance, interacted with NLRP3. TXNIP(-/-) mice and NLRP3(-/-) mice showed improvements of glucose tolerance and insulin sensitivity (53). The inhibition of TXNIP would be a potent pancreatic β cell protective agent and is a candidate for the treatment of T2DM (54). In addition, it is possible that chronic inflammation is related to the activation of the AIM2 inflammasome by aberrant glucose metabolism. Studies have shown that AIM2 expression and serum cellular mtDNA levels increased in the monocytes of T2DM patients, which might be involved in the inflammatory process in patients with T2DM (55, 56). Inflammatory signaling pathways activated during pyroptosis further exacerbate insulin resistance. Therefore, understanding the intricate relationship between pyroptosis and insulin resistance provides valuable insights into the underlying mechanisms of metabolic disorders. Targeting pyroptosis and related inflammatory pathways may hold therapeutic potential for alleviating insulin resistance and improving metabolic health.

### 3.1 Diabetic nephropathy

Diabetic nephropathy (DN) is a prevalent complication of diabetes and is associated with increased morbidity and mortality (57). Accumulating evidence demonstrated that many critical biological processes were involved in DN, including pyroptosis and subsequent inflammation (58, 59). In a streptozotocin (STZ)-induced DN rat model, ASC and caspase-1 levels were observed to increase, along with hyperuricemia and hyperlipidemia with higher proinflammatory factors levels (60). The implication of NLRP3 inflammasome activation in DN is noteworthy, as the overexpression of NLRP3 inflammasome can lead to pyroptosis (60, 61). Further research has documented that suppression of NLRP3 inflammasome activation alleviates renal injury in DN. Li Q et al. asserted that the mitigation of high glucose (HG)-induced pyroptosis in Madin-Darby canine kidney (MDCK) cells can be achieved by reducing the expression levels of NLRP3 and GSDMD (62). Moreover, the inhibition of pyroptosis by mediating NLRP3 inflammasome pathways can alleviate podocyte pyroptosis (63–65). As researchers continue to explore this field, the up-regulation of Toll-like receptor 4 (TLR4) and GSDMD coincides with the tubular injury observed in DN patients. In a high-glucose environment, treatment with inhibitors can reverse highly expressed GSDMD-NT while preventing the release of IL-1 (66). These investigations highlighted the significance of pyroptosis and the associated inflammatory response in driving the progression of DN.

### 3.2 Diabetic cardiomyopathy

Diabetic cardiomyopathy (DCM) is the most common complication in patients with diabetes, featured by cardiac hypertrophy and heart failure. Hyperglycemia, dyslipidemia, insulin resistance, oxidative stress, and inflammation collectively contribute to the pathogenesis of DCM (67). Notably, pyroptotic cell death has been observed in all DCM patients and NLRP3 inflammasome activation occurred in heart tissue (68). In DCM mice, the levels of NLRP3 and pyroptosis pathway-related proteins, as well as IL-18 and IL-1β, exhibited marked increases (69). Inhibiting NLRP3 inflammasome activation can ameliorate cardiac inflammation, pyroptosis, fibrosis, and left ventricular cardiac dysfunction (15, 70). Besides, AIM2, as a cytosolic DNA sensor, mediates the development of DCM through the caspase 1/GSDMD pathway. The silencing of the AIM2 gene alleviated cardiac dysfunction resulting from metabolic disorder and ventricular remodeling (71). Non-coding RNAs (ncRNAs), including miRNAs, lncRNAs, and circular RNAs, have also emerged as significant players in the development of DCM. The diabetes-induced circulation-associated circular RNA (DICAR) had an inhibitory effect on DCM, knockdown of DICAR enhanced DCM pyroptosis (14). A study shows that miR-30d expression levels increased in cardiomyocyte. miR-30d might repress FOXO3a expression and caspase recruitment domain (ARC) leading to cardiomyocyte pyroptosis in DCM. Notably, knockdown of miR-30d resulting in the downregulation of caspase-1, as well as the pro-inflammatory cytokines IL-1β and IL-18 (72).

### 3.3 Diabetic retinopathy

Diabetic retinopathy (DR) is a prevalent microvascular complication and represents one of the leading causes of blindness in adults. Epidemiological studies have proved that the prevalence and severity of DR increase with age and development of diabetes (73). Growing evidence shows that inflammation plays an important role in DR, inflammasomes like NLRP3 release large amounts of inflammatory cytokines, which increase retinal vascular permeability and exacerbate hypoxia, accelerating the progression of DR (74, 75). Studies depict that peripheral blood mononuclear cells and vitreous humor of patients with DR show higher expression levels of caspase-1, ASC, and pro-inflammatory factors compared with normal individuals (76, 77). Moreover, NLRP3 over-activation incites an inflammatory cascade leading to the disruption of the retinal neurovascular unit’s structure and function, ultimately resulting in vision impairment. Additionally, it is noteworthy that the hyperglycemic state stimulates GSDMD-mediated pyroptosis in DR (78). Overexpression of GSDMD-NT induces inflammation and pyroptosis in human retinal progenitor cells (HRPs). However, silencing the GSDMD gene effectively prevents HRP pyroptosis by suppressing the NLRP3/caspase-1/GSDMD signaling axis (79). Thus, further exploration of the mechanism of inflammasomes is thought to provide novel insights into the pathogenesis and clinical treatment of DR.

### 3.4 Other diabetic complications

More studies have found pyroptosis has also been associated with other diabetic complications including neuropathy and non-alcoholic fatty liver disease. Diabetic peripheral neuropathy (DPN) is among the most common complications of DM, and its severity increases over time with poor glycemic control (80). Sun Q et al. reported that TXNIP/NLRP3 inflammasome proteins, including caspase-1, and IL-1β expression levels were substantially upregulated in the DPN rats (81). Excessive reactive oxygen species (ROS) promotes inflammation and subsequently activates the NLRP3 inflammasome, which induces pyroptosis in DPN. Similar results were found in diabetes-associated non-alcoholic fatty liver disease (NAFLD) syndrome. NLRP3-mediated pyroptosis exhibited an elevation in the livers of both ob/ob and diabetic mice, and inhibiting NLRP3 in the liver protected against the progression of NAFLD (82).

## 4 Exercise on improving diabetic complications by targeting pyroptosis

### 4.1 Potential benefits of DM and its complications through exercise

Exercise training emerges as a potent non-pharmacological strategy for DM and its complications’ prevention and treatment. Exercise is widely recognized as one of the most critical therapeutic interventions, which can lead to weight loss and improve insulin sensitivity and pancreatic beta cell function (83–85). Exercise not only can improve lean body mass and lipid profile, but reduce renal injury and microalbuminuria, and ameliorates renal function in DN (86, 87). In DCM, exercise preserves endothelial function, improves antioxidant defenses, ameliorates mitochondrial dysfunction, and reduces cardiovascular mortality (88–90). Among NAFLD patients, exercise alleviated diet-induced intrahepatic lipid content, hepatic steatosis, content inflammation, and fibrosis (91–93). Moreover, higher levels of physical activity were associated with a reduced prevalence of abnormal retinal conditions (94). There is mounting evidence that pyroptosis is involved in the pathogenesis of DM and its complications. The experimental studies above have shown the beneficial effects of exercise on diabetes and its complications. However, it is unclear whether exercise slows down diabetes progression, and improves physical ability by inhibiting pyroptosis.

### 4.2 The key inflammatory factors in pyroptosis during exercise

Among these various types of inflammasomes, the NLRP3 inflammasome has been extensively studied in diverse mammalian cells and is associated with a range of autoimmune and inflammatory diseases. It is well known that reducing the expression of NLRP3 is linked to reduced inflammation and improved insulin sensitivity in DM patients (95). Exercise has emerged as a significant anti-inflammatory intervention, as it reduces the expression levels of inflammasome markers, including NLRP3 and caspase-1. Research led by Javaid HMA et al. showed that exercise suppresses NLRP3 inflammasome and promotes the anti-inflammatory reaction activation by stimulating Meteorin-like (METRNL) and the extracellular signal-regulated kinase (ERK) and p38 mitogen-activated protein kinase (MAPK) pathway in the obese mice induced by high-fat diet (HFD) (96).

Aerobic exercise represents an efficacious therapeutic approach in the prevention of DCM and alleviating cardiac pyroptosis. The NLRP3 inflammasome could be a key promoter in exercise-mediated alleviation of DCM. In the HFD-induced obesity model, aerobic exercise effectively suppressed the activation of the NLRP3 inflammasome in the left ventricles, consequently reducing expressions of NLRP3, ASC, pro-caspase-1, and IL-1β in the myocardium (97). The expression levels of P2X7R, NLRP3, and caspase-1 were significantly upregulated in the heart tissue of HFD rats. Furthermore, the expressions of the NLRP3, caspase-1and IL-1β induced by palmitic acid (PA) in H9c2 cells were significantly decreased by the P2X7R inhibitor, thereby indicating that aerobic exercise could promote cardiac remodeling by reducing inflammation and reducing P2X7R expression in HFD rats (98).

ROS trigger the NLRP3 inflammasome activation and contribute to nonalcoholic steatohepatitis (NASH) progression (99). Exercise has been shown to effectively decrease hepatic lipid content, inhibit inflammation and excessive production of ROS in the liver (100). Notably, exercise-induced increasing adropin expression was accompanied by decreased levels of ROS and NLRP3 inflammasome, suggesting that adropin may be a key role in the protection against NLRP3 inflammasome activation in NASH mice (101). In addition, regular exercise has significant potential to protect against diabetic kidney injury. Exercise improved renal function, oxidative stress, inflammation, and fibrosis in db/db mice. Aerobic exercise training decreased the levels of Nox4, ROS, TNF-α, MCP-1, IL-6, and the expression of NLRP3, ASC, caspase-1 p20, and IL-1β and IL-18. These results demonstrate that aerobic exercise exerts a renoprotective effect by inhibition of the Nox4/ROS/NF-κB/NLRP3 axis (102).

Moreover, neuronal inflammation is mainly attributed to the release of inflammasomes by NLRP3. A study found that diabetic rats exhibit significantly higher expression of NLRP3 in the prefrontal cortex, whereas aerobic exercise effectively restores NLRP3 levels to a normal state. Aerobic exercise-induced amelioration of diabetes-induced inflammation in the prefrontal cortex by inhibiting FOXO1/NF-κB/NLRP3 inflammatory signaling pathway (103). In diabetic mice, regular exercise reduces inflammasome-associated pyroptosis signaling, which prevents bone loss and improves osteogenesis. As a result of miR-150-5p’s inhibition of FNDC5 protein expression and irisin levels in STZ-induced diabetic mice models, skeletal loss and an inflammatory response occurred. Meanwhile, exercise increased the expression of FNDC5/Irisin in diabetic bones by inhibiting osteoblastic miR-150-5p and the pyroptosis-associated proteins (NLRP3, caspase-1, GSDMD). Together, exercise prevents diabetes-mediated skeletal loss and reduces cortical mechanical strength by blocking the pyroptosis pathway *via* decreased expression of miR-150 (104).

### 4.3 The clinical implications of exercise on treat DM and its complications

**Figure 2:**
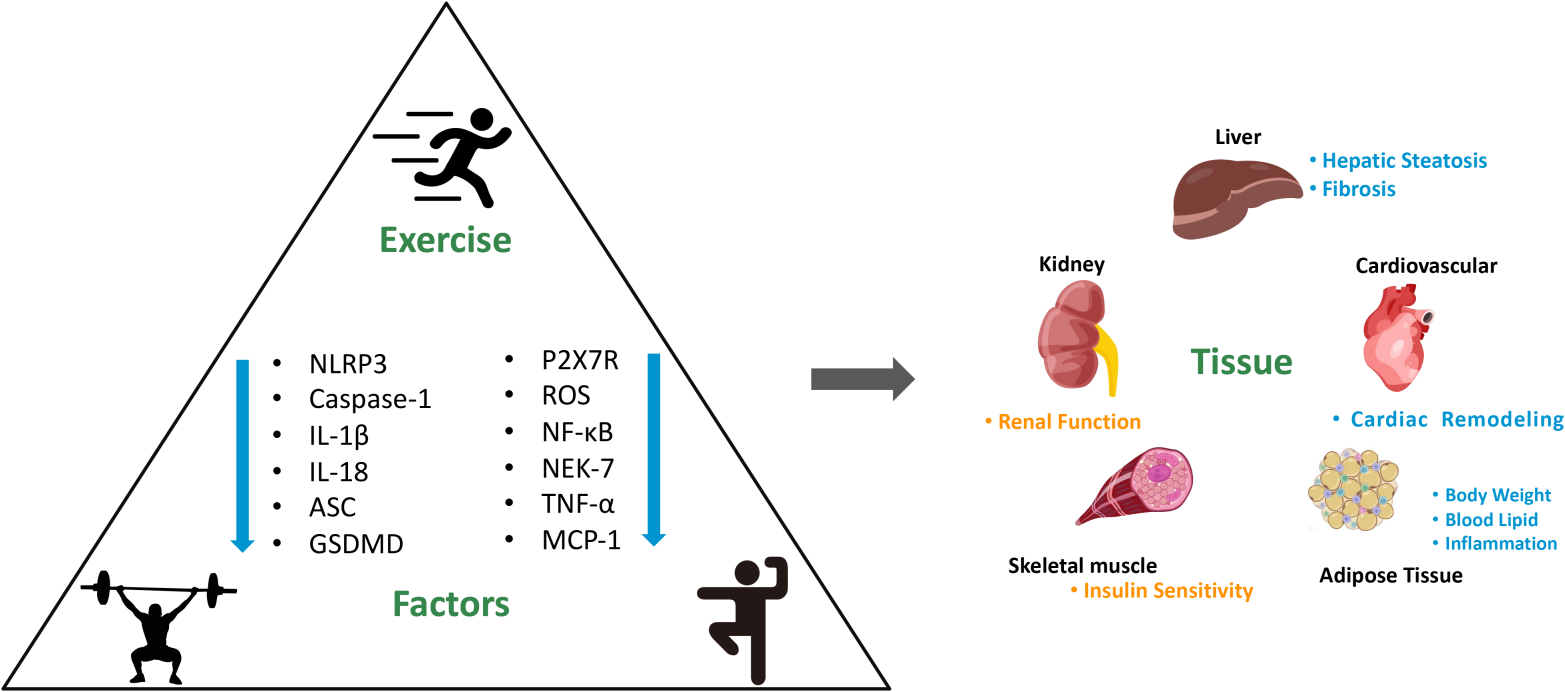
Mechanism of exercise on improving DM and its complications by targeting pyroptosis and the consequences for main organs. NLRP3, nod-like receptor family pyrin domain containing 3. GSDMD, gasdermin D. ROS, reactive oxygen species. NF-κB, nuclear factor kappa b. NEK-7, never in mitosis a-related kinase 7. TNF-α, tumor necrosis factor-α. MCP-1, monocyte chemoattractant protein-1. “↓” shows that the levels downregulated by exercise.

Effectively managing chronic diseases can profoundly influence an individual’s quality of life. The effect of exercise on treating chronic conditions is of significant physiologic and clinical importance (105). Our previous study has shown that weight-bearing running training alleviates age-related muscle atrophy by inhibiting the expression of pyroptosis-related genes in adipose tissue (106). Understanding the role of pyroptosis in chronic diseases such as DM allows for prevention and personalized treatment strategies. Evidence shows that 12 weeks of Tai Chi intervention effectively alleviated glucose homeostasis and inhibited the expression of the NLRP3 inflammatory signal pathway in middle-aged and elderly pre-diabetic patients (107). Moreover, the combined of Yijinjing and resistance training has proven effective in inhibiting the robust NLRP3 inflammasome activation, thereby alleviating insulin resistance and liver injury in elderly pre-diabetes (108). Research has also shown that exercise can exert an anti-inflammatory effect by reducing circulating levels of inflammasome activation-related inflammatory cytokines IL-1β and IL-18 in overweight/obese populations (109). Similarly, a previous study has demonstrated that a 12-week strength and endurance combined training significantly inhibited the activation of the NLRP3 signaling pathway in obese children (110) (as shown in **Supplementary Table 1** and **Figure 2**). Pyroptosis-induced inflammation is closely linked to insulin resistance and impaired glucose metabolism. Exercise training targeting pyroptosis and inflammation may lead to improving glycemic control. Surprisingly, there has been scant research conducted on the enhancement of insulin sensitivity through exercise-mediated modulation of pyroptosis. More in-depth clinical studies can fully elucidate the exact mechanisms for the efficacy of exercise in regulating pyroptosis and alleviating insulin resistance.

## 5 Conclusion and prospects

Pyroptosis is one of the prominent forms of programmed necrotic cell death. Current studies strongly show that pyroptosis plays a vital role in the progression of various diseases, including CNS disorders, immunological diseases, atherosclerosis, and cancer. While recent studies have uncovered the molecular mechanism underlying pyroptosis activation in diabetes remains elusive. Thus, finding new treatments and intervention mechanisms to target inflammasomes and inhibit the pyroptosis signaling pathways is necessary for DM and its complications’ treatment. In this review, we concisely summarize the potential role of pyroptosis in diabetic complications, elucidating the underlying pathophysiological mechanisms. Additionally, we highlighted the important role of the protective effect of exercise on DM and its complications by blocking the pyroptosis-associated inflammasome pathway. Exercise training could suppress NLRP3, caspase-1, NF-κB, ROS, P2X7, IL-1β, and IL-18, the pyroptosis-associated inflammasome pathway is primarily contributing to this effect. However, there are still some problems that need to be solved. The underlying mechanisms of the exercise on other inflammasomes and pyroptosis pathways in DM and its complications remain limited and have challenges to both experimental and clinical investigations. Moreover, the distinct effects of various exercise patterns on pyroptosis-associated mechanisms need further elucidation. Thus, more in-depth *in vitro* and *in vivo* studies will be necessary to explore the efficacy of exercise in regulating pyroptosis-induced cell death and inflammasomes in DM progression and may be providing valuable insights for the treatments of DM and its complications.

## Author contributions

NL and LZ wrote the original manuscript and draw the figure. XW sorted out the literature. YZ and LG reviewed and revised the manuscript. All authors contributed to the article and approved the submitted version.
