# Supplementary material for: Exploring exercise-driven inhibition of pyroptosis: novel insights into treating diabetes mellitus and its complications
Source: Front Endocrinol (Lausanne). 2023 Oct 4;14:1230646. doi: 10.3389/fendo.2023.1230646 (PMC10582706; doi:10.3389/fendo.2023.1230646)
Supplement: Supplementary file 1 [file Table_1.docx]

Supplementary Material

# Supplementary Table

Table 1. Effects of exercise on pyroptosis-related factors in different diseases.

| **Diseases** | **Object** | **Exercise pattens** | **Effects of exercise** | **Functions** | **Reference** |
| --- | --- | --- | --- | --- | --- |
| HFD (high fat diet)-induced Obesity | C57BL/6 mice  3T3-L1 mouse pre-adipocytes | Treadmill training (8 weeks) | NLRP3⭣, caspase-1⭣, IL-1β⭣, and IL-18⭣ | Attenuating fat accumulation and insulin resistance, suppressing inflammation in adipose tissues | (96) |
| HFD-induced Obesity | Sprague-Dawley rats  H9c2 myocytes | Treadmill training (12 weeks) | P2X7R⭣, NLRP3⭣， caspase-1⭣, and IL-1β⭣ | Reducing body weight, blood lipid concentrations, inflammation; reversing the cardiac remodeling | (98) |
| Obesity | human | Strength and endurance combined training (12 weeks) | NLRP3⭣, Caspase-1⭣ | Reducing plasma glucose levels and increasing dynamic strength, inhibiting inflammatory stat | (110) |
| Pre-diabetes mellitus | human | Tai Chi intervention (12 weeks) | ROS⭣, NF-κB⭣, NEK7⭣, NLRP3⭣, ASC⭣, Caspase-1⭣, gasdermin D (GSDMD)⭣, IL-1β⭣, and IL-18⭣ | Reducing body weight and blood pressure, reducing the expression of serum inflammatory factors | (107) |
| Pre-diabetes mellitus | human | combined Yijingjing and resistance training (6 months) | NLRP3⭣, cleaved caspase-1 ⭣, cleaved IL-1β⭣, IL-1β⭣ | Improving insulin resistance, reducing the chronic inflammation | (108) |
| Diabetes | Sprague–Dawley rats | Treadmill training (4 weeks) | NF-κB⭣, NLRP3⭣ | Inhibiting inflammation and improving synaptic plasticity | (103) |
| Type 2 diabetic mellitus (T2DM) | C57BL/6J mice | Treadmill training (8 weeks) | NLRP3⭣, caspase 1⭣, and GSDMD ⭣, miR-150, IL-1β⭣, and IL-18⭣ | Improving trabecular bone microarchitecture and bone mechanical properties; ameliorates diabetes-associated glucose intolerance and bone loss | (104) |
| Diabetic Cardiomyopathy (DCM) | C57BL/6 mice | Treadmill training (20 weeks) | NLRP3⭣, ASC pro-caspase-1⭣, and IL-1β⭣ | Attenuating cardiac remodeling and mitigating pyroptosis | (97) |
| Nonalcoholic Steatohepatitis （NASH） | C57BL/6 mice | Treadmill training (12 weeks) | NLRP3⭣, caspase-1⭣, IL-1β⭣, and ROS⭣ | Alleviating diet-induced hepatic steatosis, inflammation, and fibrosis | (101) |
| Diabetic kidney disease (DKD) | db/db mice | Treadmill training (8 weeks) | Complex I⭣, MDA⭣, Nox4⭣, ROS⭣, TNF-α⭣, MCP-1⭣, IL-6⭣ and IL-18⭣; p-NF-κB⭣, p65⭣, and I κBα⭣, NLRP3⭣, ASC⭣, caspase-1 p20⭣, and IL-1β⭣. | Reducing body weight and microalbuminuria, improving renal function, and attenuating renal pathological changes | (102) |

Note: “↓” shows that the levels downregulated by exercise.
